# Subject Positioning in the BOD POD® Only Marginally Affects Measurement of Body Volume and Estimation of Percent Body Fat in Young Adult Men

**DOI:** 10.1371/journal.pone.0032722

**Published:** 2012-03-26

**Authors:** Maarten W. Peeters

**Affiliations:** Department of Kinesiology, Research Center for Exercise and Health, Katholieke Universiteit Leuven, Leuven, Belgium; Universidad Europea de Madrid, Spain

## Abstract

**Introduction:**

The aim of the study was to evaluate whether subject positioning would affect the measurement of raw body volume, thoracic gas volume, corrected body volume and the resulting percent body fat as assessed by air displacement plethysmography (ADP).

**Methods:**

Twenty-five young adult men (20.7±1.1y, BMI = 22.5±1.4 kg/m^2^) were measured using the BOD POD® system using a measured thoracic gas volume sitting in a ‘forward bent’ position and sitting up in a straight position in random order.

**Results:**

Raw body volume was 58±124 ml (p<0.05) higher in the ‘straight’ position compared to the ‘bent’ position. The mean difference in measured thoracic gas volume (bent-straight = −71±211 ml) was not statistically significant. Corrected body volume and percent body fat in the bent position consequently were on average 86±122 ml (p<0.05) and 0.5±0.7% (p<0.05) lower than in the straight position respectively.

**Conclusion:**

Although the differences reached statistical significance, absolute differences are rather small. Subject positioning should be viewed as a factor that may contribute to between-test variability and hence contribute to (in)precision in detecting small individual changes in body composition, rather than a potential source of systematic bias. It therefore may be advisable to pay attention to standardizing subject positioning when tracking small changes in PF are of interest.The cause of the differences is shown not to be related to changes in the volume of isothermal air in the lungs. It is hypothesized and calculated that the observed direction and magnitude of these differences may arise from the surface area artifact which does not take into account that a subject in the bent position exposes more skin to the air in the device therefore potentially creating a larger underestimation of the actual body volume due to the isothermal effect of air close to the skin.

## Introduction

Air displacement plethysmography (ADP), commercially available in the BOD POD ® -system [1] has become a relatively widespread method for determining body volume, which in combination with body mass can be used to calculate body density (BD). In a two-component model of body composition at the molecular level [2] , where body mass is divided into fat mass and fat-free mass, BD determined by ADP or hydrostatic weighing can be used to estimate percent body fat (PF) assuming a constant composition of fat-free mass [3], or body volume can be used in multi-component models to obtain a more detailed picture of an individual's body composition (e.g. [4]). Apart from some potential technical imprecision [5] the main sources of measurement error or variability in ADP seem to be related to the sources of isothermal air which have to be controlled or adjusted for in order to correct the raw body volume (BVr) measure of the BOD POD ® to the actual body volume (BV) of the subject. Not controlling or adjusting the BVr obtained by ADP causes an underestimation of the subject's BV since isothermal air is more compressible than the air under adiabatic conditions in the test chamber of the device [1]. This underestimation of the BV would result in an overestimation of BD and an underestimation of PF. Four sources of isothermal air have been recognized. The first one is isothermal air trapped in clothing. Deviating from the prescribed minimal clothing, i.e a tight fitting swimming suit, has repeatedly been demonstrated to cause substantial bias which seems to increase as the amount of clothing and hence isothermal air increases [6]–[11]. Isothermal air in the scalp hair has to be limited by providing thorough compression by wearing a swim cap [12], [13] which preferably also has to be tight fitting [13] and it has been suggested that body hair may be an issue as well [14]. If the first two sources of error are controlled for by standardizing the attire, the BVr is corrected to obtain the actual body volume (BV) of the subject by the formula:
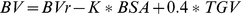
(1)In which BSA is the body surface area calculated based on height and weight by the Dubois & Dubois [15] formula and K is a constant which is approximately −4.67*10^−5^. TGV stands for the thoracic gas volume at mid-exhalation which can either be estimated or preferably is measured using the BOD POD ® system after the BVr measurement [16]. K*BSA is known as the surface area artifact (SAA) and adjusts for the isothermal air close to the skin. Using a different formula to calculate BSA only resulted in a bias of approximately 0.1%fat which is very small [17].

It has been shown that the BOD POD ® can reliably and accurately measure the volume of inanimate objects [1] in which isothermal effects are not an issue although there is some evidence the precision is less in volumes below 40 liters [17]. Therefore most of the potential errors or bias when measuring human subjects will most likely not arise from technical imprecision but from the potentially inadequate controlling or correction for the sources of isothermal air. One factor that may affect the adequate correction for isothermal air is subject positioning in the BOD POD®. Subject positioning has been suggested to potentially be a source of variability or error in repeat measures of ADP [5], [18]. However, to our knowledge the potential effect of subject positioning in ADP has not yet been investigated, If subject positioning affects the measured body volume in a subject this will automatically influence the technical error of measurement (TEM) and hence contribute to test-retest variability if the subject position is not standardized. If longitudinal follow-up of a subject and detection of relatively small changes in body volume, body density and % body fat, is the aim of the measurement, one would want to minimize the technical error of measurement (TEM) by carefully controlling all factors influencing measurement variability.

The hypothesis of the present study is that the raw body volume will be larger when subjects sit in a more forward bent position as compared to when they sit up straight with the shoulders to the rear. The proposed mechanism would be that the TGV at mid-exhalation is hypothesized to be smaller when subjects are sitting in this forward bent position and hence its effect on the raw body volume measurement will be smaller such that the raw body volume will underestimate the actual body volume to a lesser extent. To correct for this potential bias in the raw body volume due to subject positioning the TGV at mid-exhalation would have to be measured with the subject sitting in the same position in which the raw body volume measurement was done. Using an estimated TGV would therefore not be adequate to correct for this hypothesized bias due to subject positioning.

## Materials and Methods

30 young adult men were recruited via flyers among the K.U. Leuven university student population. All procedures were approved by the Medical Ethics committee at the K.U.Leuven and participants provided written informed consent prior to participating. Measurement protocol.

Stature was measured using a wall-mounted Harpenden stadiometer with subjects on bare feet and the head positioned in the ‘Frankfurt plane’. Body mass was measured to the nearest gram and rounded to the nearest 0.1 kg using the electronic Tanita®-scale attached to the BOD POD®. Subjects wore only their swim suit during weight measurement. The scale was calibrated on a daily basis.

Body composition was measured by air-displacement plethysmography using the BOD POD® following the manufacturer's guidelines [1] except for the subject's position (see below). All subjects wore tight fitting speedo-type swimsuits and wore a silicon swim cap to provide optimal compression of the scalp hair [13]. As the hypotheses was based on the fact that TGV may change due to the position of the subject and therefore may affect the amount of isothermal air in the lungs and hence the correction applied to the raw body volume measurement extra attention was paid to familiarizing the subjects with the procedure to measure the TGV using the BOD POD® system. Therefore before the actual testing procedure subjects were allowed to practice the procedure used to measure the TGV several times with a maximum of 10 attempts. If two consecutive measurements had a merit <1.0 and an ‘airway’ <35 ml as prescribed by the manufacturer and were within a range of 250 ml the actual testing procedure was started. An error of 250 ml in TGV results in an ‘error’ of 100 ml in actual body volume which is similar to acceptance criteria for measurement of the residual lung volume in hydrostatic weighing, which also results in an error in body volume of 100 ml. In total 26 subjects met these criteria during practice trials. Four subjects could not produce acceptable merit and –or airway values repeatedly and were excluded from further analysis. One subject who met all criteria produced physiologically impossible values of <1.0 liters for TGV and was also excluded from further analyses. Descriptive statistics of the final sample (n = 25) can be found in table 1.

**Table 1:** Descriptive characteristics of the subjects (n = 25).

Age (years)	20.7±1.1 (18.0 to 22.7)
Stature (cm)	182.3±6.5 (171.3 to 2.00)
Weight (kg)	75.8±7.4 (67.9 to 94.5)
BMI (kg/m^2^)	22.6±1.4 (19.3 to 25.2)

Values are mean ± standard deviation (range).

**Figure 1:**
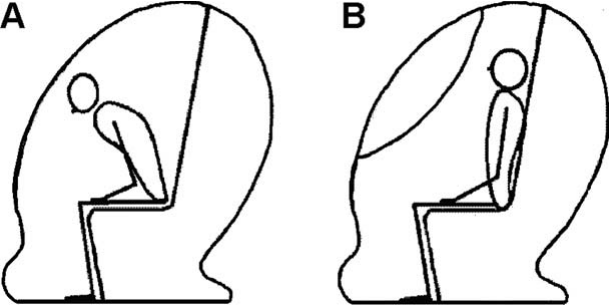
Subject positioning in the BOD POD®. A: Bent forward position; B Sitting up straight.

The BOD POD operators manual (LMI PN #2102946, Rev E, 01/05/04) states that the subject has put his/her hands in his/her lap and ‘sit comfortably’, however the body composition of the subjects in the present study was measured twice in a forward bent position with shoulders hanging and curved back (fig. 1A) and twice they were requested to ‘sit up straight’ with shoulders to the rear (fig. 1B). These two positions were chosen in an effort to maximize the potential effect of subject positioning such that ‘sitting relaxed’ as requested in the operator's manual would most likely represent a position that is intermediate to both ‘extreme’ positions represented in figure 1. The four measurements were conducted in random order to avoid test-order effects and were completed within a time-span of approximately 30 minutes. The mean results of the two straight-up measurements and the mean of the two ‘bent’ measurements were used in further calculations comparing the effect ‘between conditions’. In each condition the ‘measured thoracic gas volume’ was used to correct for the isothermal air in the lungs. Subjects were instructed to assume the same position during lung volume measurement as during the raw body volume assessment: i.e. ‘bent’ or ‘straight-up’. The raw body volume obtained from each procedure was corrected with the surface area artifact and the measured TGV to obtain the actual body volume (BV) from which then total BD was calculated. Conversion of BD to PF was done using the Siri equation [3].

In our lab a CV of 0.0457% was found based on for 40 measurements of the 49.381 L calibration volume over a period of six weeks [9]. For single repeated measurements within day TEM using ADP in our facility is 76 ml for raw body volume, which corresponds to a TEM of 0.57%fat using a predicted thoracic gas volume [9].

### Analyses

Paired t-tests and regression analysis were used to compare the body volumes, measured TGV and PF between both conditions. Bland Altman plots [19] were used to evaluate the mean bias, limits of agreement and to explore potential trends in bias. All analyses were done using SAS 9.1 (SAS Carey Inc.). Significance was set at p<0.05.

## Results

**Figure 2:**
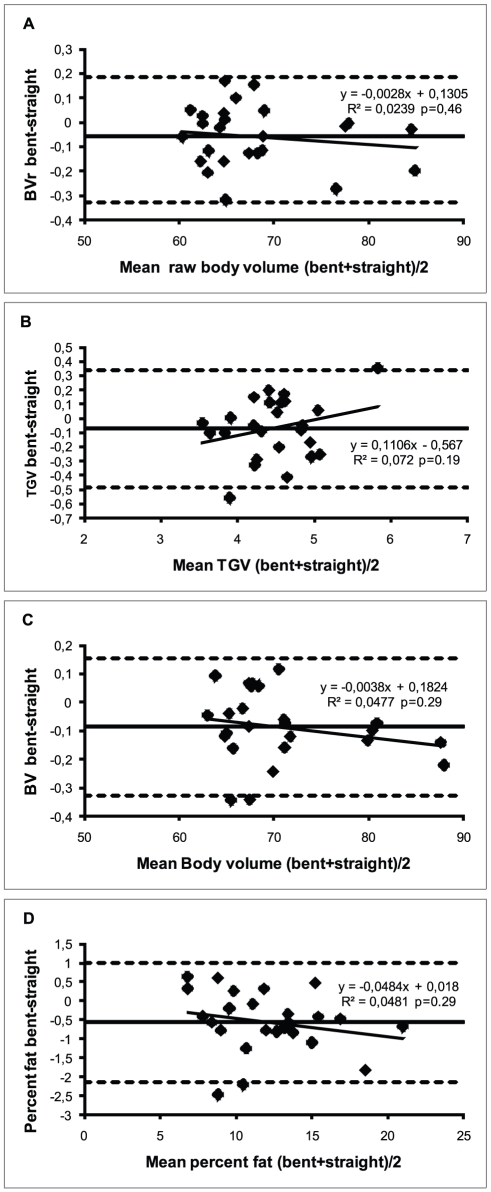
Bland Altman plots for raw body volume, thoracic gas volume, body volume and percent fat. BVr = raw body volume (panel A), not corrected for isothermal effects of the surface area artifact and of the thoracic gas volume; TGV = thoracic gas volume at mid-exhalation (panel B); BV = actual body volume (panel C), corrected for the isothermal effect of the surface area artifact and of the thoracic gas volume; percent fat (panel D) was calculated from body density using the SIRI formula. All panels include mean bias (boldface solid line) ±1.96 times the standard deviation of the mean bias (interrupted lines) and linear regression (thin solid line) to evaluate significance of potential trends in mean bias.

Mean raw body volume, measured TGV, body volume and PF are shown in Table 2. The raw body volume and the corrected body volume were significantly (p<0.05) smaller in the bent position resulting in a lower percent body fat in the bent position (p<0.05), compared to the straight position. The TGV did not differ significantly between both subject positions (p<0.05). Bland-Altman plots did not show any significant trends in mean bias (Fig. 2 A,B,C, D). R^2^ from regression analysis were above 0.99 for raw body volume and actual body volume, 0.96 for PF, and 0.85 for measured TGV. Only for measured TGV slope and intercept deviated significantly from 1.0 and 0.0 respectively (p<0.05).

**Table 2:** Differences in raw body volume, measured thoracic gas volume, body volume and percent fat in both subject positions.

	Bent forward	Straight	Difference
Raw body volume	68.009±6.893 L	68.067±6.912 L	−58±124 ml*
Measured TGV	4.445±0.549 L	4.517±0.494 L	−71±211 ml
Body volume measured TGV	70.708±7.026 L	70.795±7.053 L	−86±122 ml*
Percent fat measured TGV	11.8±3.6% fat	12.3±3.7% fat	−0.5±0.7% fat*

Values are mean ± standard deviations; TGV = Thoracic Gas Volume;

* p<0.05 paired t-test.

## Discussion

The results of the present study suggest that subject positioning has a small but significant effect on the estimated PF using air-displacement plethysmography such that resulting PF is on average 0.5%fat higher when subjects are requested to sit up straight compared to when the same subjects are sitting in a more bent position with shoulders hanging and back curved (Fig. 1 A).

It was hypothesized that raw body volume would be larger in the bent position compared to the straight position. The proposed mechanism was that by sitting in a bent position the actual lung volume would be reduced and hence there would be less isothermal air in the lungs. Isothermal air is a cause of underestimating actual body volume due to its higher compressibility than air under adiabatic conditions. The error in raw body volume would persist in the actual body volume if the estimated TGV would be used to correct for the isothermal effect of the TGV, as this is simply based on height, age and gender and therefore independent of the subjects position. If the TGV was measured in the in same position, either bent or straight, the difference in raw body volume would be corrected and result in no significant differences in actual body volume and hence PF. However, there was substantial variability in the difference scores in measured lung volume between both extreme positions and therefore the mean difference of −71 ml albeit in the hypothesized direction was not statistically significant. Given the fact that only 40 percent of the measured lung volume has to be added to the raw body volume measurement the mean adjustment due to isothermal air in the lungs is only −28 ml in our subjects. Furthermore the observed difference in raw body volume was in the opposite direction of what was hypothesized, such that adjusting the raw body volume for the measured lung volume actually increased the mean difference between both conditions from −58 ml in the raw body volume to −86 ml in the corrected actual body volume (table 2).

The observation that the actual effect on the raw body volume of sitting in a bent position vs sitting up straight is opposite of that which would be expected under the proposed hypothesis suggests that there is another source of variation that causes the observed effect. One may speculate that the effect of decreased isothermal air in the lungs is masked by a larger opposite effect. However all subjects were allowed plenty of practice trials to familiarize them with the procedure of measuring TGV and produce reliable results. Furthermore the mean of two measurements was taken as the actual TGV in both positions. Given the variability and non-significance of the difference in measured lung volume in both positions it can be concluded that difference in actual TGV is unlikely to be a measurable and actual cause of substantial differences in raw body volume.

Although the resulting difference in PF between both positions is fairly small, it is consistently observed in the same direction in 80% of the subjects (fig. 2D) and has a standard deviation of 0.7%fat which is similar to the standard deviation of differences observed between two consecutive measurements in the straight position (SD = 0.8%fat, results not shown). It has been shown that the BOD POD canreliably measure the volume of inanimate objects, in which effects of isothermal air are of no concern [1], [9]. Furthermore the position of the subjects in the present study was randomized to exclude test-order effects. Therefore the remaining cause of difference between both positions is likely to be related to the sources of isothermal air. The subjects wore the same swimsuit in all positions, wore the same swim cap in both conditions and difference in TGV was shown not to be significant. Therefore the only source of isothermal air that may be affected by the subject's position is the surface area artifact. The correction of the raw body volume by the SAA is intended to correct for the effect of isothermal air close to the skin and hence does not take into account the position of the subject and therefore at first glance cannot be a source of variation. However a difference may arise when more skin is exposed to the air in the BOD POD in one position than in another: if a subject sits in the ‘bent’ position, his back does not touch the seat of the BOD POD, such that a larger portion of the skin is exposed to the air in the BOD POD and hence may cause an isothermal effect resulting in a smaller raw body volume. When a subject is requested to sit up straight at least a portion of his back touches the seat thus eliminating the isothermal effect of the air close to that portion of the skin. To see if the order of magnitude of the observed effect is consistent with the hypothesized explanation of our results one may consider the following example: our subjects are on average 183 cm tall and weigh75 kg which for this ‘average subject’ results in a mean BSA of 19658 cm^3^ using the Dubois & Dubois [15] formula. Surface area of the trunk in the Dubois and Dubois paper is calculated by the formula 0.703*L(M+N) in which L is the length of the trunk, measured from the suprasternal notch to the upper border of the pubes, and M is the waist circumference at the level of the umbilicus and N the circumference of the thorax at the level of the nipples. Assuming L = 52 cm, M = 80 cm and N = 100 the trunk of our average subject has a surface area of 6580 cm^3^ or approximately one third of the BSA. Assuming 25% of the trunk touches the seat since the front and sides cannot touch the seat and the back is unlikely to be in full contact, 1645 cm^3^ of the skin would not be exerting an isothermal effect in the straight position, which corresponds to 8.4% of the BSA. Since the SAA is a linear transformation of the BSA the actual surface area artifact would be reduced by 8.4% as well when subjects are in the straight position compared to the bent position. In our ‘average’ subject this would result in an ‘error’ in the actual SAA from −0.918 to −0.840 L which is a difference of 78 ml and is in the order of magnitude of the differences observed in the present study. This mean difference due to changing subject positioning, which can presumably be attributed to the SAA, is five times larger than that caused by using alternative formula's to calculate BSA which was reported to be around 0.1%fat by Collins et al [17]. If this proposed mechanism indeed is correct and a subject has to assume a bent forward position due to being very tall or the subject cannot sit with his back touching the seat due to for example ambulatory problems the test leader may want to bear in mind that the body volume of the subject may be slightly underestimated compared to when part of the subject's back indeed is touching the seat. Similarly when measuring young children, the back of the subject may also not be in contact with the seat because their legs are not long enough to both sit with bended knees and have their back against the support. Measurement of body composition by ADP should be undertaken with awareness of those factors that may cause variability in the result [17] and the present study suggests that subject positioning may be one of those factors contributing to this measurement variability. Therefore if a subject changes from one extreme position to another in a longitudinal follow-up of for example an elite athlete small changes in body volume may either be masked or increased depending on the direction of the actual changes and the change in subject position. The TEM calculated based on two single BOD POD procedures in the present study was 88 ml and 91 ml in the straight and the bent position respectively. If subject position between the two consecutive measurements is changed between the two single BOD POD procedures this TEM increased by about 30% to 115 ml (results not shown). This corresponds to an increase in TEM from 0.55% fat to 0.75%fat. Given the fact that the observed body volume lies with 95% certainty within the range of the ‘true’ value ± 2*TEM, (the ‘true value’ is. the value that would be obtained by the device if no measurement variability exists) this would imply that observed changes in percent fat larger than 1.1% fat are likely to reflect ‘true’ changes in percent fat if subject position is monitored. If subject positioning is not monitored this may increase to 1.5% fat in the ‘worst case’ scenario, if the subject changes from one extreme position to the other. If the repeated measures are based on the average of two full BOD POD procedures, in all likelihood this confidence interval will be decreased in accordance with the classical test theory and changes of perhaps less than 1%fat may be detected reliably by air displacement plethysmography which is similar to what has been reported for hydrostatic weighing [20], [21]. The effect of subject positioning on measurements by ADP found in the present study should be viewed in the context of reducing between-trial measurement variability, rather than a potential source of systematic bias such as those resulting from using different clothing schemes or swim caps [6]–[10], [13]

In summary the reported mean differences due to changing the subject's position are small, especially considering that two extreme positions were imposed on the subjects. When attempting to monitor changes in body composition subjects are unlikely to change between both extreme positions. Nevertheless, standardizing instructions regarding the subject's position in the device may decrease measurement variability and hence improve the detection of small changes in body composition by air displacement plethysmography.
